# An Analysis of the Factors behind Rural Residents’ Satisfaction with Residential Waste Management in Jiangxi, China

**DOI:** 10.3390/ijerph192114220

**Published:** 2022-10-31

**Authors:** Bo Zhou, Fang Qi, Muhammad Faraz Riaz, Tariq Ali

**Affiliations:** 1 School of Economics and Management, Jiangxi Agricultural University, Nanchang 330045, China; 2 Department of Economics, Government College University Faisalabad, Allama Iqbal Road, Faisalabad 38000, Pakistan

**Keywords:** waste management, rural, end-user, China

## Abstract

With the increasing environmental and health problems caused by residential solid waste (RSW), upgrading waste disposal services has become a key priority in rural areas of developing countries. Waste disposal services can be improved by incorporating the end-user evaluation of the services and the infrastructure. This study aims to analyze the respondents’ satisfaction with waste disposal services and infrastructure in rural China, which has not been well documented in the previous literature. For this purpose, we applied the ordered probit model on survey data of 1064 rural residents of Jiangxi, China. In two separate models, two independent variables, i.e., users’ ranking of waste disposal management services and waste disposal management infrastructure, were regressed on five sets of policy, personal, social and demographic, environmental, and village characteristics of the respondents. Our results show that rural residents have relatively high satisfaction (level four out of five) with RSW services. We found a significant correlation between all five investigated characteristics (personal, social and demographic, environmental, and village characteristics) and respondents’ satisfaction with RSW management. However, the correlation differs in magnitude and direction among different respondent groups, where gender, minority status, the sanitary condition of household toilets, and treatment of toilet waste at the village level have the largest influence on satisfaction. It was found that male respondents, ethnic minorities, residents with non-farming status, and respondents with more sanitary household toilets have higher satisfaction levels. Our results provide crucial references for decision-makers to effectively promote the further optimization and improvement of rural waste disposal systems in the future.

## 1. Introduction

Over the recent decades, waste pollution has substantially endangered human health and quality of life and has affected the way we set development targets [1,2,3,4,5]. Despite spending 20% of their local budgets on waste management, 90% of waste is openly dumped or burned in low-income countries [6]. In China, rural waste management is directly related to the fundamental well-being of 600 million rural residents and the environmental quality of more than 90% of the country’s land area. Accompanied by rapid industrialization and improved living standards, China’s rural areas generate increasingly higher volumes of solid waste. In 2021, rural areas generated ~520 million tons of solid waste, compared to 230 million tons for urban areas [7]. Rural solid waste has grown by ~8–10% annually in recent years [8]. Depending upon the region, kitchen waste (30–41%), inert waste (18–63%), plastics (3–14%), and paper, wood, and glass (3–22%) make up a large proportion of rural waste in China [9].

China prioritizes rural waste management to improve the rural human settlement environment and ecological revitalization [10] and strengthen rural infrastructure and service networks [11]. Different operational and management methods for waste management are adopted in various places, including county-wide mode, market-oriented service outsourcing operation and management mode, inter-village joint construction and self-operation management mode, semi-market operation management mode, and purchasing rural property management mode [12]. Rural domestic waste treatment modes can be divided into four types: the on-site centralized treatment mode, urban-rural integrated treatment mode, decentralized household treatment mode, and decentralized plus urban-rural integrated treatment mode. Domestic waste treatment technologies widely used in China include landfill, composting, and incineration [13]. There are ten standards for waste classification in China, including one national standard, two industry standards, and seven local (provincial) standards [14].

Although several reforms are already underway in urban waste management, waste collection, transportation, treatment, and disposal efforts in rural China still lag far behind [15,16]. About 40% of rural RSW cannot be disposed of innocuously [17]. Poor waste management in rural areas has caused air, soil, and water pollution in these localities and can potentially cause serious health risks for the residents [18]. Research on rural waste management in China mainly focuses on rural residents’ willingness to participate in governance [19,20], willingness to pay, and cooperative behavior on waste classification [21,22], classification intentions, and behavioral differences [23,24].

Disposing of an increasing amount of waste is a significant environmental and economic challenge, especially in rural areas. Sustainable and effective waste management strategies can be better developed if government departments and other stakeholders have a better understanding of the preferences and attitudes of the masses regarding waste management. Previous literature has highlighted the importance of research focusing on the quality of service provided by solid waste treatment providers [25,26,27,28]. Some studies have shown that end-users usually prefer the private sector providing solid waste collection services over the public sector due to the better service quality of the former [29,30,31]. Guerrero et al. [32] showed that coordination and cooperation between service users and providers are critical for willingness to pay, participation in solutions, and interest in solid waste management. Previous literature on waste collection services in China focuses more on technical and operational aspects. The end user’s satisfaction with these services is mostly ignored or analyzed in urban settings.

It is also recognized that both public and private service providers consider user satisfaction as one of their leading performance measures [33]. Evaluation of user satisfaction can highlight challenges and opportunities regarding the provision of these services. Planners, managers, and operators can reduce service costs and improve service delivery through informed decisions.

The assessment of customer satisfaction helps in the easy identification of prevailing and emerging operational challenges as well as efficient aspects. This further helps in planning and taking proactive managerial and operational measures to improve customer satisfaction, reduce costs, maximize profit, and improve customer experience. Several studies have investigated the influence of waste pollution on individuals’ satisfaction, including in developed countries [34,35,36] and developing countries [37,38]. Globally, several studies have analyzed the relationship between residents’ satisfaction with waste management, such as [39] for Nigeria, [40] for Malaysia, [41] and for Spain.

Caputo et al. [42] analyzed key factors affecting the perception of water contamination and its risky consumption from a community clinical psychological perspective in El Salvador. They found heterogeneous perspectives about water quality and sanitation in the study area. Yao et al. [43] employed a regulated model to analyze the impact mechanism of air pollution perception on young talent urban settlement intentions in Hangzhou, China. They showed that air pollution perception significantly impacts young talent’s urban settlement intentions, where residential satisfaction acts as the intermediary effect. Wang et al. [44] explored the impact of service quality on tourists’ safety perception in the urban forest along with the specific reasons for the effect in Fuzhou, China. Their results indicated that visual quality and traffic accessibility positively affected tourists’ safety and control perceptions, respectively. Safety emotion and control perception were positively related to facility completeness. They concluded that improving the service quality of urban green spaces can improve tourists’ perception of the safety of the urban forest environment.

For China, some studies have been conducted on respondents’ satisfaction with livability and its determinants [45,46]. Chu et al. [47] used a binary logistic regression model on data from 469 households in Harbin, China, to investigate factors affecting residents’ satisfaction with municipal household solid waste treatment performance. They found that ‘pick-up frequency by ‘waste collection vehicles’, ‘publicity and education’, ‘fund supply situation’, and ‘charging standard for waste treatment’ were the four principal determinants of respondents’ satisfaction with waste treatment. Ao et al. [48] used factor analysis and a logistic regression model to analyze the indicators influencing rural residents’ satisfaction with rural household latrines in Sichuan, China. They found that respondents are relatively satisfied with rural household latrines, and their satisfaction is affected mainly by the basic condition of latrines, village committee performance, transparency of village affairs, sources and subsidies of funds, construction participants, and construction methods.

However, to the best of our knowledge, no study has analyzed the determinants of end-users’ satisfaction with government-provided waste disposal services in less-developed rural areas of China. It is still unclear what factors determine users’ satisfaction with waste management services in these areas. In light of this research gap in the literature, this study’s primary goal is to better understand users’ satisfaction with waste management services in rural settings. Specifically, we aim to highlight the personal, social and demographic, environmental, policy, and village characteristics of the end-users related to waste management services that may give rise to different levels of satisfaction with these services. The end-user satisfaction with public services in waste management can be a helpful guide for implementing and improving effective waste management systems in developing countries, particularly in the rural areas that are lagging in this respect.

We have designed this study to fill this research gap by focusing on China’s relatively less-developed rural area in the southern province of Jiangxi. We use the ordered probit technique on a questionnaire-based survey data of 1064 respondents from 108 villages of Jiangxi province. The study aims to answer the following research questions. (1) What personal, social, and environmental factors of respondents affect respondents’ satisfaction with local authorities’ management services of RSW and waste collection and disposal infrastructure? (2) Are there any collective (village level) determinants of respondents’ satisfaction with local authorities’ waste disposal management services (WDMS) and waste collection and disposal infrastructure (WCDI)? This study provides an overall picture of how rural users view waste management services and how their satisfaction levels differ due to various characteristics in rural Jiangxi. The findings are crucial for the authorities in the process of designing and providing effective and specific action plans for Jiangxi province in particular and China’s rural areas in general.

This study contributes to the literature in the following aspects: (1) in general, it estimates the satisfaction of rural residents with waste management services in rural areas and critical factors affecting the satisfaction, which, to the best of our knowledge, has not yet been attempted in the literature; (2) in particular, it uses a newly available large dataset of an economically backward province (Jiangxi) to showcase the lower bound of residents’ appraisal of waste management services in rural China; (3) in light of the finding, it suggests important policy recommendations for improving waste management.

The rest of the paper proceeds as follows. Section 2 describes the method and data used in this study. Results are presented in Section 3. Section 4 contains a discussion of the results. Section 5 presents the conclusions and policy implications.

## 2. Methods and Data

### 2.1. Theoretical Framework

**Figure 1:**
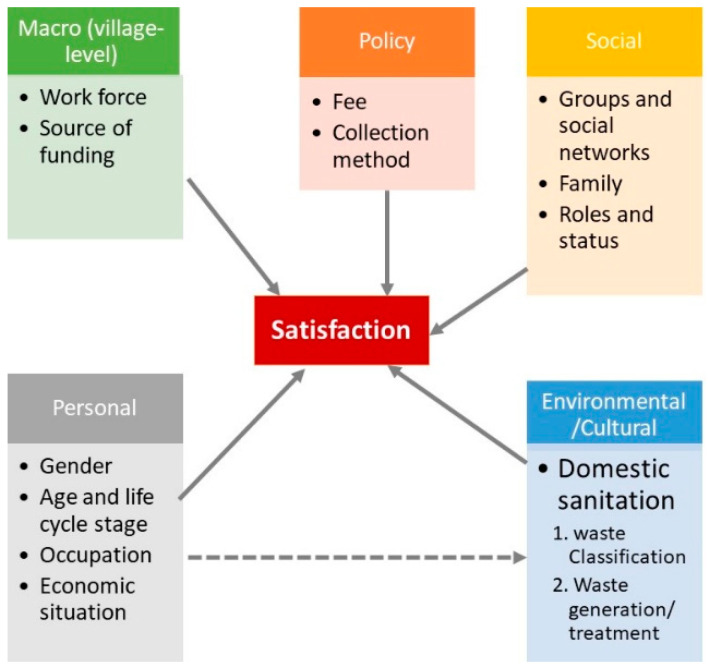
Theoretical framework of satisfaction with rural waste management services (partially adopted from [49]).

End-users adoption and participation in public services such as waste management can be thought of as a process similar to consumers’ choice of goods and services, which can be affected by personal, social, cultural, and psychological characteristics (see [49] (p. 159) for a thorough discussion of consumer buying behavior). In addition to these factors, site level (village level in this case) factors and policy environment may also affect how users perceive these services (see Figure 1). It may not be possible for the service provider, e.g., local government, to consider all such factors while designing and implementing waste management services; they can still keep these factors in mind.

*Personal factors:* People’s tastes and preferences vary according to gender and age, e.g., young females might be more demanding of waste management services than older females or even males due to the former’s deeper engagement in household chores and exposure to waste management activities in daily life. Several studies have regarded gender as a key personal characteristic affecting a person’s decisions and actions toward environmental responsibility [50,51,52]. According to gender socialization theory, our behaviors are often affected by gender expectations under cultural norms [50]. Male and female children learn different values and face divergent social expectations even from early childhood [53], so much so that gender differences may turn into a critical foundation for gender-based dissimilarities in our environmental intentions [53]. Therefore, we expect gender to affect satisfaction with waste management services significantly.

In a review of 58 studies that investigated the relationship between reported satisfaction and age, Crow et al. [54] (2002) found that older respondents were significantly more satisfied in 41 (70.7%) studies. This could be related to generational or life-cycle effects, i.e., older people are more stoical and accepting than the young, or they engender more respect and care from their providers. Alternatively, it may be a cohort effect, and they have lower expectations based on prior experiences when standards were lower, as seen in Crow et al. [54].

A person’s occupation affects the perception of services they receive in their locality. For example, due to the rugged nature of their jobs, blue-collar workers may be more easily satisfied with waste management services than white-collar workers. People with higher incomes may have higher expectations and may show lower satisfaction for the same services than people with lower incomes. A person’s lifestyle is not just their social class or personality; it captures their whole pattern of acting and interacting in the world [49].

*Social factors:* Several social factors also affect a user’s perception of public services, such as the consumer’s small groups, social networks, family, and social roles and status. For example, ‘membership groups’ to which a person belongs can have a direct influence, and ‘reference groups’ can act as a reference in shaping one’s attitudes or behavior. Personal words of family, friends or other users can profoundly impact people’s perception of public services rather than what the government tells them. End-user opinions can be influenced by ‘opinion leaders’ due to the latter’s more profound knowledge, improved skills, or other characteristics. People usually show appreciation for products/services that correspond to their roles and status in a family, organization, or even society. For example, minority respondents may report different levels of satisfaction due to the difference in their expectations of public services or less understanding by service providers of cultural norms and expectations of users of different backgrounds. Hence, if these users’ expectations are not met, they may show lower satisfaction with the service [55].

*Environmental/cultural factors:* A person’s behavior is fundamentally formed by the culture, where people learn perceptions, wants, and behaviors from their family and other important institutions in their society. For example, the cultural shift toward greater concern about a healthy environment has boosted the demand for improved waste management services. Even ‘subcultures’ in society, e.g., those with minority status, may exert varying influences on how people perceive public services in a locality. People of the same ‘social classes’—upper, middle, lower—are likely to share similar values and show identical behaviors. People with similar orientations towards generating and managing household waste may show similar perceptions about public waste management services. Expectations are based on information that users collect from various sources, such as personal experiences, cultural norms, media influences, and the views of close ones. Individuals with deeper interest and exposure to public issues deepen their knowledge of service standards and may shape their expectations, and such individuals may be less satisfied with services of the same standard. Access to information about alternatives that raise expectations may lower satisfaction with no change in the service standards [54].

*Policy factors:* The charges (if any) paid by the users and methods used to collect and treat household waste may act as a ‘policy environment’ that shapes people’s attitudes towards waste management services. The service charges may act as price, and users paying this price may have higher expectations for the waste management services. It is shown in the literature that institutions that formulate and implement policies (rules etc.) can also change users’ satisfaction levels [47,56].

*Macro (village-level) factors:* The overall socio-economic development and environmental status of a site (village, in this case) can influence people’s perception of waste management services. For example, people belonging to a more developed site and with environmental standards are expected to be more demanding towards waste management services. People living in a village with more external linkages may also be tougher on local management services. Previous literature has also shown that users in more developed (urban) regions report lower satisfaction than the ones living in less developed (rural) regions [57].

### 2.2. Survey Design

**Figure 2:**
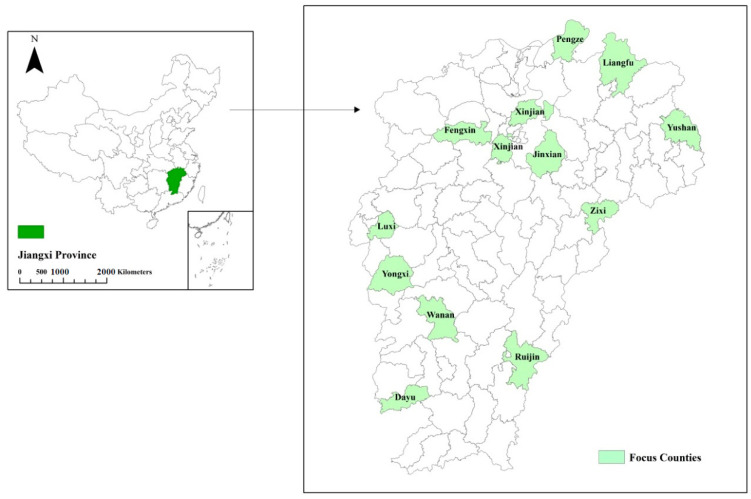
Study area in Jiangxi Province.

The data used in this study came from the ‘Data Platform Construction Project for Think Tanks of China’s Rural Revitalization Strategy’ jointly carried out by the School of Advanced Agricultural Sciences, Peking University, Beijing, and Jiangxi Agricultural University, Nanchang, in December 2019. We designed the stratified random sampling procedure and final survey instrument with the village as the unit of analysis. The fieldwork team of six researchers and eighty graduate students/research fellows chose the sample and implemented the survey in Jiangxi provinces and 12 counties in a provincially representative sample. Survey teams implemented a uniform process in each sampled county to select sample villages. First, the sample selection involved choosing the counties, towns, and villages. Twelve counties were selected from Jiangxi province, one from each quintile of per capita Gross Value of Industrial Output (GVIO) (Figure 2). GVIO was used because it is one of the best predictors of both standards of living and development potential and is often more reliable than net per capita income. The survey team chose three townships within each county, one township with a per capita GVIO above the county median and one with a per capita GVIO below the county median.

Secondly, three villages were chosen within each township, using the same procedure to select townships. The survey collected information at the village level in December 2019.

The village head facilitated the data collection and provided information on three critical stages of the RSWM process: waste collection, transportation, and disposal.

In order to determine the most recent state of waste collection services, we asked the village head to describe whether or not waste collection facilities and equipment (e.g., dumpsters, garbage pits, or garbage storage buildings) had been built in their village and whether or not the village committee was employed for this service. The survey teams also asked whether each village transported waste to a disposal facility. In addition, we also collected village and township characteristics. Village characteristics included net per capita annual income, the number of small hamlets (the number of groups within each village), total population, and distance from towns (the distance from the village committee to the township government).

Each village leader was first approached to get the village residents’ lists. A village committee member accompanied the survey team to each household to introduce the survey’s objectives to the residents. The data collected on printed structured questionnaires through face-to-face interviews with household heads were then entered into computer sheets and processed through Stata. The questionnaire has two main parts, i.e., the household and village sections. The household section comprises 19 sub-sections ranging from the socio-economic background, e-commerce, technical training, household credit, sewage and waste management, and rural governance. The village section contains information on solid waste management, toilet waste treatment, toilet revolution, public infrastructure, labor force, livestock farming, etc. The questionnaire was improved after a pre-survey pilot. Out of 1080 rural households, 1064 households were used in this study after eliminating outliers and missing values. The sample size was determined following [58], which shows that for a large population (>1 million), a sample of 384 can provide satisfactory results. We have used a sample of 1064 respondents in this study, which is much larger than the suggested sample size.

The study area (Figure 2) consists of counties in different parts of Jiangxi province. The province is located in the middle and lower reaches of the Yangtze River, with abundant rainfall and sufficient sunlight. There are plain basins for rice planting, hills suitable for cash crops, and economic forests. The overall per capita disposable income is 28,016 RMB, ranking 14 among all regions in China, and the rural per capita disposable income is 16,981 RMB, ranking 10. Jiangxi’s agricultural sector accounts for 14.1 of the provincial GDP% [59]. The provincial HDI is 0.74, with the 19th ranking in China [60].

Most of the villages are the Han majority and have a village leadership that controls and governs the day-to-day affairs of the village. These areas have a relatively wet climate with hilly topography. Mining is the most important profession for many residents, after agriculture. Of the 108 villages covered in this study, 100 had at least a basic waste management system funded chiefly by central and provincial governments. The central parts of Jiangxi province are urban counties and were thus not covered in this study.

The time period covered the critical phase when Jiangxi was in its initial phases of adopting ‘improved waste management’ in its rural areas through several policy-level changes, including the National Toilet Revolution Project. The government launched these changes during 2015–2018 and aimed to improve the sanitary infrastructure for rural users while implementing better waste and sewage treatment [61]. Using survey data from this period can bring new insights for policymakers.

### 2.3. Econometric Model

When the dependent variable of a regression model is in discrete form, a discrete choice model should be chosen, such as a binary choice model, a multi-valued choice model, and a ranking and counting model [62,63,64]. In this study, the two dependent variables, i.e., satisfaction with RSW disposal management services and satisfaction with public waste collection and disposal infrastructure, were in discrete form over five levels, with 5 indicating very satisfied and 1 indicating very dissatisfied. As the nature of our dependent variable is discrete in nature (which means that a respondent has to categorically choose one of the choices from 1 (very dissatisfied) to 5 (very satisfied)), naturally, the choice of estimation technique would be one of the discrete choice models. The binary probit is used among discrete choice models when the dependent variable is dichotomous. However, in our case, the dependent variable is not dichotomous and contains more than two choices which have a natural ordering (5 (very satisfied) is one extreme, and 1 (very dissatisfied) is the other extreme, and as the scale moves from 1 to 5 it gradually adds higher levels of satisfaction).

In the case where the multinomial-choice variable is in ordered form, although the outcome is discrete, the usual multinomial logit or probit models cannot explain the ordinal nature of the dependent variable. On the other hand, ordinary regression analysis (OLS) would have the opposite problem. For example, if the response variable in an opinion survey is coded as 0, 1, 2, 3, or 4, linear regression would treat the difference between a 4 and a 3 the same as that between a 3 and a 2, which would be wrong, because they describe the ranking of choice [65] (p. 736). Therefore, using the normal usual multinomial logit or probit models or the linear regression model would not do justice to our data, which is in an ordered form. The ordered probit model has been widely used for the analysis of ordinal data (see [66,67,68,69,70,71]).

Therefore, we used the ordered probit model to analyze the relationship between the key independent and dependent variables. In statistics, ordered probit is a generalization of the widely used probit analysis to the case of more than two outcomes of an ordinal dependent variable (a dependent variable for which the potential values have a natural ordering, as in poor, fair, good, excellent).

**Figure A1:**
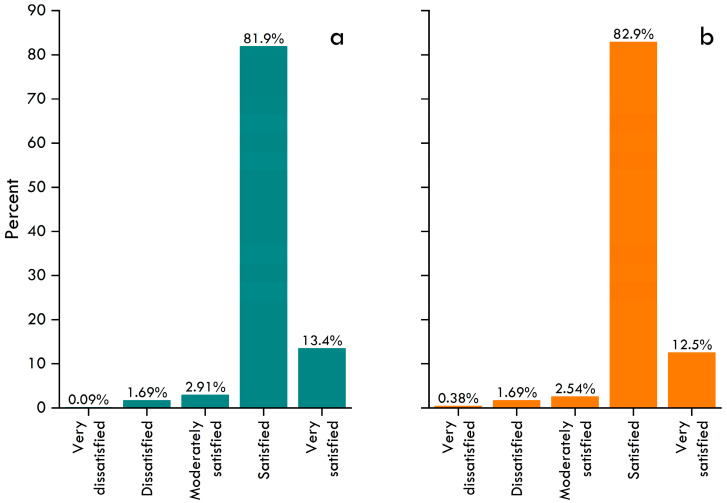
Satisfaction level of respondents (**a**) with waste disposal management services; (**b**) with waste collection and disposal infrastructure. *Source*: variables are based on our survey data.

Following Greene [62], a basic model can be written as follows:

(1) $$\begin{array}{c} \begin{array}{c} \begin{array}{c} Y_{i}=1\\ Y_{i}=2 \end{array}\\ \ldots \end{array}\\ Y_{i}=5 \end{array}\{\begin{array}{c} ifY^{*}\leq \alpha_{1}\left(=1\right),\\ if\;\alpha_{1}\leq Y^{*}<\alpha_{2},\\ \ldots \\ if\;\alpha_{4}\leq Y^{*\;}. \end{array}\;$$

where $Y^{*}$ is a continuous implicit variable and $\alpha_{1}$, $\alpha_{2}$, $\alpha_{3}$, $\alpha_{4}$ are the cut points. The respondent is very dissatisfied when $y^{*}$ is below the critical value $\alpha_{1}$. Similarly, when $y^{*}$ is higher than the critical value $\alpha_{4}$, the respondent is assumed to be very satisfied. Although the value of $y^{*}$ cannot be observed, the respondent’s answer to his/her satisfaction level can be obtained. The specific ordered probit models used in this paper can be written as follows:

(2) $${\mathrm{Satisfaction}}_{1ij}=\alpha_{1}WP_{ij}+\alpha_{2}X_{i}$$

(3) $${\mathrm{Satisfaction}}_{2ij}=\alpha_{1}WDM_{ij}+\alpha_{2}X_{i}$$

where ${\mathrm{Satisfaction}}_{ij}$ are the dependent variables, where ${\;\mathrm{Satisfaction}}_{1ij}$ shows respondents’ satisfaction with waste disposal and ${\;\mathrm{Satisfaction}}_{2ij}$ shows their satisfaction with waste collection and disposal infrastructure. $WP_{ij}$ is our first core variable showing whether or not a respondent has to pay a fee for disposing of his/her waste.$\;WDM_{ij}$ the second core variable of our interest, indicating various waste disposal methods adopted by respondents. $X_{I}$ indicates a set of other explanatory variables used in this study (Appendix A Figure A1 and Table A1).

### 2.4. Selection of Independent Variables

Various factors influence our satisfaction with RSW disposal management services and the collection and disposal infrastructure financed and managed by the government in our areas. Satisfaction depends not only on the internal factors of households but also on the external environment. Therefore, based on some important studies in this field (such as [37,44,45,47,48,72,73]) and our sample data, we added the independent variables to our econometric models based on their relevance to our dependent variables.

The variables used in this study have also been employed in similar studies in this field. For example, Wilson et al. [74] suggested that the fees/charges for household waste treatment may affect residents’ satisfaction. For example, Evison and Read [75], Haight [76], and Deng et al. [77] advocated the effect of education on residents’ satisfaction with waste treatment. Improved government policies and regulations on waste management can also improve customer satisfaction [78,79]. Koo et al. [80] found a significant effect of the waste bins deployment on residents’ satisfaction with the treatment of household waste. Doberl et al. [81], Athimulam and Odayar [82], and Khan and Farooqi [83] suggested a link between government investment in waste treatment and residents’ satisfaction with the performance of the waste treatment services. Collection frequency of sanitation vehicles was found as a determinant of residents’ satisfaction with waste treatment [84,85].

In this paper, the first core variable for satisfaction with RSW collection and disposal infrastructure is ‘waste payment’, where respondents were asked, “do you have to pay for disposing of the RSW?”. This variable shows whether the respondents share any costs related to waste disposal management (WDMS) in their village or whether the government bears all the costs. The second core determinant of satisfaction with RSW disposal is ‘waste disposal methods’, divided into four categories. These categories include: ‘Littering: respondent throws away waste at any random place’ (Disposal Method 1) and ‘Door-to-door collection: the cleaners come to the respondent’s doorsteps to collect the waste’ (or Disposal Method 2). Then, waste Disposal Method 3, i.e., Method 2 + Storing, combines ‘Door-to-door collection’ and ‘Storing: waste is placed in public waste bins, public waste storage buildings, or public waste pits’. Finally, waste Disposal Method 4 is ’Storing: waste is placed by residents in the public waste bins, public waste storage buildings, and public waste pits.’

We have categorized other explanatory variables into four categories. Firstly, the basic characteristics of the respondent include the household head’s gender, education, age, annual net income, skills training, and profession. The second set of variables includes information related to social status, which is external to the respondents. For example, ethnicity is a dummy variable, with ‘1’ representing Han Chinese (the majority) and ‘0’ representing non-Han Chinese respondents (the minority). Party membership (Communist Party of China) is a dummy variable, with those who answered ‘yes’ coded as ‘1’ and those who answered ‘no’ coded as 0. Several types of hukou (registered permanent residence) are practiced in rural China: non-farmer resident households are coded 1, while farmer households are coded 0.

The third set of explanatory variables relates to the environment, such as ‘whether or not the respondents classify their waste?’. Participation in the Toilet Revolution Project, the respondents were asked, “did you participate in the national Toilet Revolution Project when you built your toilet?’. Household toilet sanitation was measured by a five-point Likert scale, ranging from ‘1’ to ‘5’, with ‘1’ showing very unhygienic and ‘5’ showing very hygienic. Involvement in livestock raising was determined by whether or not the household raised livestock, poultry, or other animals (not including pets) in 2019. Daily fecal discharge of livestock was measured by converting the daily waste emissions per animal into daily waste emission units (grams/day), using conversion methods by Laguë [86] and Spiehs and Varel [87].

The fourth set of explanatory variables includes information related to the village of each household. For example, the number of workers returning to villages from outside the province (taken as the number of return migrants in a year); the number of workers returning from other cities in this province (taken as the number of return migrants in a year); the number of workers returning from outside counties in the same city (to which this village belongs, taken as the number of return migrants in a year); the primary source of funding for the Toilet Revolution Project in the village (dummy variable with government funding = 1, self-financing = 0); whether or not the village disposes toilet waste after proper treatment (Yes = 1, No = 0); time that the village Party Secretary and Village Director spend on dealing with village affairs (number of hours/day); maintenance cost of water conservancy infrastructure paid for by village funds (RMB/Average year); the wage of male workers (RMB/day), and wage of female workers (RMB/day).

Although theoretical relationships point towards causal links between dependent and independent variables in our analysis, this might be an association due to the cross-sectional nature of our data. Still, we have a strong theoretical conviction that if the time period for the data is expanded, the relations between the variables will persist.

## 3. Results

Overall, the sample respondents show relatively high satisfaction with both waste disposal management services and infrastructure (Appendix A Figure A1). A small proportion (27.2%) of the respondents paid some fee for RSW disposal. Most (76%) of the respondents use public waste bins, storage buildings, or pits to dispose of their RSW.

The regression analysis mentioned above was carried out using the software “Stata”. First, all the data were cleaned and transformed into various variables used in this study. Moreover, to check for the validity of our regression analysis, we calculated the Variance Inflation Factor (VIF) of the regression. The value of VIF was less than 10 showing the absence of a multicollinearity problem in our data, which means that our independent variables are not highly correlated, allowing us to interpret the results confidently.

### 3.1. Influence of Personal Characteristics

The main results for ordered probit models for satisfaction with RSW disposal management services (WDMS, in Model 1) and waste collection and disposal infrastructure (WCDI, in Model 2) are shown in Table 1. Firstly, in addition to the core variables ’waste payment’ and ‘methods of waste disposal’, the ‘personal characteristics’ of the respondents are shown, such as gender, age, education, income, skills training, workplace, and profession. Our results of Model 1 show that paying a fee for waste management does not affect respondents’ satisfaction with the services (WDMS, −0.118). However, the payment negatively influences respondents’ satisfaction with infrastructure (WCDI, −0.289 *) in Model 2.

Respondents who disposed of their RSW via the channel ‘Door-to-door collection plus Storing’ were less satisfied with WDMS (−0.430 *). Similarly, respondents who chose the fourth type of waste disposal channel (Storing) had even lower satisfaction with WDMS (−0. 614 **) than with the former method.

Among the personal characteristics, males are typically more satisfied with WDMS (0.262 *) and WCDI (0.180 *) provided by the local authorities than females. Similar to coefficients with several other variables, the coefficient’s size is small; in statistical terms, it still shows a difference between males and females. Skills training lowers respondents’ satisfaction with the WCDI (−0.303 **). Respondents working in their county of permanent residence are less satisfied with WDMS (−0.186 *) in Model 1. Because these respondents use public services and encounter related issues more frequently than those who work outside the county and spend a shorter time in their village, their satisfaction with WDMS is lower than those who have work and spend more time outside the county. Furthermore, as shown in Table A2, only around 45% of the villages have at least one toilet; this low percentage may be one of the reasons for the dissatisfaction among residents. On the contrary, there is no apparent difference between the satisfaction with WCDI for respondents working in or outside the county.

Of occupational aspects, public servants are less satisfied (−0.438 ***) with WCDI because they know more details about WCDI in their village, and after comparing it with more developed places such as cities, they think that the government can do better in WCDI in their villages. Self-employed respondents also have lower satisfaction with the WCDI (−0.265 **, Model 2) than farmers, industry, or manual laborers. Since the self-employed respondents have lived and operated their businesses in rural areas for a long time, they pay attention to and keep in mind the development of the waste collection and disposal infrastructure and have higher expectations and requirements for the construction of WCDI.

### 3.2. Influence of Social and Demographic Characteristics

The demographic characteristics of the respondents are covered by ethnicity, party membership, and hukou, of which the ethnic majority (ethnicity = Han Chinese) are less satisfied with WDMS (−0.841 *, Model 1) and WCDI (−1.220 **, Model 2). It is also worth mentioning here that the coefficient (−1.220 **, Model 2) is greater than one because we report the betas, not the probabilities, from the ordered probit model results. Betas can be greater than one and even negative. Respondents with a Party membership, and political identity in China, show lower satisfaction (−0.165 **) with WDMS than non-member respondents.

Regarding hukou, non-farmer respondents have lower satisfaction with WDMS (−0.169 **, Model 1) and WCDI (−0.205 **, Model 2) than respondents with farmer hukou. The coefficients’ size shows that influence is stronger (more negative) for infrastructure than management services

### 3.3. Influence of Environmental Characteristics

Among the environmental characteristics, better sanitary conditions of household toilets can raise respondents’ satisfaction with WDMS (0.260 ***, Model 1) and WCDI (0.167 ***, Model 2). This is an interesting outcome showing how personal choices in terms of better environment and health conditions can shape one’s perception of the public services they use in their daily lives.

### 3.4. Influence of Village Characteristics

Among the village-related (collective) characteristics, respondents have low satisfaction with WDMS (−0.007 ***, Model 1) and WCDI (−0.005 ***, Model 2) when the village has a higher number of workers returning after working outside the province. The results also show that respondents feel more satisfied with WDMS (0.170 *) and WCDI (0.217 **) if they live in a village where the primary funding for the Toilet Revolution Project comes from higher-level financial appropriation than if villagers mainly assimilate the funding through collective funds in the village. Respondents who live in villages where the village authorities use more environmentally friendly methods to dispose of household toilet waste are more satisfied with WDMS (0.233 **) and WCDI (0.290 ***). This is perhaps because when the rural respondents see a more pro-environmental attitude from village authorities regarding toilet waste, they feel better about the RSW disposal services provided by the same authorities.

Moreover, the longer the village Party Secretary and Director spend time dealing with village affairs, the higher the respondents’ satisfaction with WDMS (0.016 ***) and WCDI (0.016 ***) in their village. It is akin to respondents’ visual perception of how their leaders’ hard work can improve the public services in their areas. At the same time, bearing the maintenance cost of water conservancy infrastructure by the village authorities plays a vital role in the local environment. Therefore, the more the village government spends on this infrastructure, the higher the respondents’ satisfaction with WCDI (4.87 × 10^−^^6^ **). In addition, the wage of male workers during the busy and slack season is also positively associated with satisfaction with WDMS (0.002 ***, Model 1) and WCDI (0.002 ***, Model 2).

It is worth mentioning here that usually, the outlier problem arises in continuous variables. Most of our data were in discrete choice form, which significantly lowers the chances of outliers in the data. Still, to ensure our analysis’s consistency, we ran our analysis using both with and without outliers (there were a few outliers in the dependent variables). The comparison of these results showed no significant differences.

## 4. Discussion

This study used survey-based data from rural respondents to analyze factors affecting their satisfaction with waste management services in China. Overall, satisfaction with waste management services is quite high in the region. The reason for high satisfaction could be improved services in the region than before [88,89].

Paying a fee for the waste management infrastructure is negatively associated with users’ satisfaction. The results are similar to the findings of [90] but opposite to those of [91]. This finding can be related to the post-purchase process, where the payees are less satisfied than the respondents who do not pay. Moreover, this result can also be associated with the overall financial conditions of the sampled villages (see Table A2); as the average income in the sampled villages is not so high, the respondents may feel it is quite a burden to pay for waste-related services. The low level of satisfaction of the respondents who pay for the disposal services could be due to the low quality of the infrastructure [9,92] compared to its fee, which is more visible than the management services. Respondents using ‘Door-to-door collection plus Storing’ or ‘Sorting’ methods reported lower satisfaction levels, possibly because (1) they are not happy with the frequency/timing or even the attitude of the waste collectors, (2) by opting for a more environmentally friendly option of putting their waste in the government-installed facilities, they feel that even though they try to contribute their share by bringing the waste to public waste facilities, the government-provided WDMS is still lagging. These results align with the findings of [90], Table A3, which showed that ‘waste separation behavior’ in some areas can lower residents’ satisfaction.

In China’s rural areas, women are more frequently involved in issues related to RSW than men [93,94]. Therefore, females frequently encounter the government’s arrangements for RSW disposal and are more likely to identify shortcomings in rural solid waste management. Similar studies have also shown that females are engaged in recycling activities because they are usually more involved in performing domestic tasks [95] and have to bear a greater burden of recycling tasks than men in a household [96].

Respondents’ skill training can lower their satisfaction with the WCDI, which aligns with our expectations, as the training broadens the respondents’ horizons, not only in terms of general knowledge of life but also in agriculture and agroecology, and environmental sustainability [97]. Consequently, the respondents’ newly acquired perspective on the environment, including waste collection systems, raises their expectations, making them less satisfied with WCDI than respondents without skills training.

Another key finding is that public servants have a lower satisfaction level with WCDI, which is similar to several other studies. In this regard, Wang et al. [98] discussed that public servants not only have a better sense of social responsibility but can also appraise environmental, social, and economic situations better. The outcome of our mode is similar to that of Huang and Du [99], who showed that public servants’ satisfaction with public housing in Hangzhou, China is lower than people working in the private sector.

Another intriguing result of our analysis is that respondents belonging to minority groups show higher satisfaction, possibly due to their lower expectations and the special treatment the minorities receive under various government policies. Several previous studies also have similar findings on China. For example, Song et al. [73] showed that satisfaction with public services positively correlates with the ratio of the ethnic minority to the total population in a town. These respondents find their current situation is better than in the past, so they have higher satisfaction, even though they have lower public service quality than surrounding areas. Zhang et al. [100] and Xie and Zhao [101] found that minority rural residents are more satisfied/happier than the Han majority.

Being a party member can lower the user’s satisfaction with WDMS. This suggests that party members are more likely to critically judge the basic rural environment due to their frequent participation in party activities. At the same time, they are exposed to other better WDMS from outside-the-village places through their higher ranks, so they feel less satisfied with the WDMS in their villages. Existing literature shows that being a party member in China positively influences the “environmental awareness” of the individual [102].

Hukou—belonging to the farmer or non-farmer type residence status—is an important socio-economic measure in China. Our findings show that non-farmers have low satisfaction because non-farmer residents have more chances of witnessing the urbanization process than the farmer residents (who lack a direct comparison perspective); as the former have more opportunities to work and live in cities, they could experience improved waste disposal services and develop a better understanding and expectations. The lack of direct comparison has also been found as one of the reasons for farmers’ higher satisfaction with public services in China [73].

Respondents living in houses with better sanitary conditions are more satisfied with waste management services. The reason could be that respondents who keep their household toilets clean are also more concerned about the environment. Several studies have shown that engaging in pro-environment behavior is positively associated with subjective well-being and/or life satisfaction [103,104,105,106,107,108]. Moreover, a common assumption is that increasing personal concerns about the environment will increase pro-environmental actions [109]. Swaim et al. [110] demonstrated that respondents’ attitudes play an important role in teaching them about environmental sustainability, whereas attitudes include respondents’ engagement in environmental sustainability at home, like recycling, etc.

The village-related (collective) characteristics can also shape respondents’ perceptions of waste management services. For example, our results indicate that satisfaction drops in villages that have more returning workers from outside the province. Jiangxi is ranked low in development status in China; migrant workers returning from outside the province are usually exposed to better public services, including good waste disposal services practices. On their return, they will most likely communicate their experiences accumulated throughout their sojourns with their village fellows, and thus, the respondents become less satisfied with their waste disposal practices. Additionally, the average distance of the sampled villages from highways is not high (see Table A2), so the villagers may frequently visit other relatively developed places that may raise their expectations. Literature suggests that return migrants can bring change in the collective action of their home communities by bringing new ideas, norms, and practices from the outside world [111,112,113]. In Chinese villages, information is shared and reproduced through outdoor gatherings, covering a wide array of topics related to the life of the village [114].

We also found that respondents’ satisfaction improves in villages that use more provincial/central funding for environmental/health projects. This indicates respondents’ desire for the government to bear the cost of sanitation projects and is consistent with other studies in the field [46]. The National Toilet Revolution Project is a national-level program launched in early 2018 by China’s government. It promotes constructing and renovating sanitary toilets for rural users and implementing waste and sewage treatment [61]. Another finding is that respondents’ satisfaction with the waste disposal services improves when they see their leaders strive harder to provide public services to the village community. These results show that respondents’ satisfaction with waste disposal services in their village cannot be separated from government work efficiency and methods. The literature also suggests that public services are the most important of all dimensions of the influencing factor on respondents’ overall satisfaction with rural livability [46].

Respondents living in villages with a higher wage of male workers also have higher satisfaction. Probably the higher income of males is because there is a labor shortage in the village or the scale of agriculture is increasing, i.e., the village is developing and becoming more non-agricultural. These factors indicate that the village has a relatively better economic situation and thus spends more resources on waste disposal services. The latter makes the residents of such villages more satisfied with WDMS and WCDI. Zhang and Zhao [94] suggested that villagers’ willingness to invest is crucial in constructing sanitation infrastructure in China’s rural areas. Village residents mainly provide these funds, although it is sourced from the state, collective, individual (private), and other funds. Therefore, higher incomes may motivate village residents to invest more in local public infrastructure.

## 5. Conclusions

Reforming public services, improving citizen satisfaction with these services, and maintaining or restoring the public trust in government have been at the heart of numerous administrative reforms. No study has analyzed the determinants of users’ satisfaction with waste disposal services in China’s rural areas. Considering the above gap in the literature, this study was designed to explain users’ satisfaction with waste management services in rural settings. Using a questionnaire survey-based dataset of 10,864 rural respondents in Jiangxi Province on self-reported satisfaction, this study finds that rural users have high satisfaction with residential solid waste (RSW) disposal and collection and disposal infrastructure.

The results of the ordered probit model show that respondents show lower satisfaction with waste collection and disposal infrastructure (WCDI) when they pay for the waste disposal services, which could be due to the low quality of the infrastructure [9,92]. Respondents who dispose of their waste in the facility provided by the government are less satisfied with waste disposal management services (WDMS). Respondents belonging to the male group, ethnic minorities, farmer hukou, and have cleaner household toilets are more satisfied with WDMS and WCDI. Being a member of the CPC lowers satisfaction with WCDI.

The gender-based difference in respondents’ satisfaction with waste management, in which males were more satisfied, was contrary to some management studies showing that male respondents generally give lower ratings than their female counterparts [115]. A possible explanation for our results can be explained based on the observation of a previous study showing that 66% of all females are “feeling” types, whereas 66% of all males are “thinking” types [116]. For our study, if one assumes that users’ satisfaction rating was based on an objective process, then the “thinking” types (males) would be more satisfied than the “feeling” types (females).

Respondents who work for the government and those working in the self-employed economy have a worse impression of waste collection and disposal infrastructure than respondents who make their living from agriculture and those working in industry or manual labor. Village-related factors such as the pro-environmental treatment of toilet waste, more funding for environmental projects from the government, and village leaders’ dedication to public services also increase people’s satisfaction with RSW. At the same time, more investment and sharing of maintenance costs (maintenance cost of water conservancy facilities) by the government are positively associated with the respondents’ satisfaction. We also find that the association of different factors with respondents’ satisfaction varies for WDMS and WCDI, where some factors have a stronger association in one model, and others are not (statistically) associated at all.

Our results can be used by policymakers to improve waste management services in rural China. The quality and effectiveness of waste management infrastructure should be improved so that end-users can contentedly pay for waste management services. Waste collectors should be given further professional training so that their service delivery standards can match rural residents’ expectations. Keeping in view the lower satisfaction of female respondents, the waste management system should be tailored to meet the needs of female residents. Currently, the services and infrastructure seem to fall short of the expectations of those who have more relevant information (party members, government servants) or have seen/can compare with better systems (people working outside the province, return migrants, non-farmer hukou), which should be taken into consideration while implementing future reforms in the rural waste management system. This also implies that the government needs to guide the public to establish a correct understanding of the waste management system so that rural residents appreciate and participate in effective waste management practices, particularly those who still lack this knowledge. The adoption of more pro-environmental initiatives, such as the active participation of local leadership in service provision, toilet revolution, and innocuous toilet waste disposal, should be promoted to improve the public trust and participation in public services. It is also crucial to encourage rural residents with different socio-economic backgrounds to participate in the planning and construction process concerning waste disposal and collection in rural areas.

It should be noted that we used a cross-sectional data set; however, the impact of time and other aspects on satisfaction with rural public services can be better captured using a longitudinal design. Future research in this field should include analysis over time using panel data. A panel data set could reveal the causal links between the variables in future studies. Moreover, the study sample consisted of rural residents from Jiangxi Province only. In future studies, this should be extended to include a national sample of rural residents’ satisfaction with overall public services in rural areas. An interesting study of user satisfaction and adoption of waste management services should include the recently adopted ‘waste classification’ initiative in many rural areas of China. We do not report rejection/failure to accept any hypotheses, as this study did not develop any particular hypotheses. The reason was the exploratory nature of the study.

## Figures and Tables

**Table 1:** Results of ordered probit models of satisfaction with RSW management services.

Variables	Waste Disposal Management Services, WDMS (Model 1)	Waste Collection Disposal Infrastructure, WCDI (Model 2)
**Policy variables**		
Waste payment *(Yes = 1, Otherwise = 0)*	−0.118 (0.144)	−0.289 * (0.158)
Door-to-door collection of waste *(Yes = 1, Otherwise = 0)*	−0.541 (0.334)	0.619 (0.620)
Door-to-door collection + Storing of waste *(Yes = 1, Otherwise = 0)*	−0.430 * (0.245)	0.538 (0.497)
Storing of waste *(Yes = 1, Otherwise = 0)*	−0.614 ** (0.270)	0.450 (0.645)
**Personal characteristics**		
Gender *(Male = 1, Female = 0)*	0.262 * (0.150)	0.180 * (0.101)
Age *(Number of years)*	0.003 (0.003)	0.002 (0.003)
Education *(Number of years of education)*	0.005 (0.012)	0.002 (0.013)
Skill training *(Yes = 1, No = 0)*	−0.058 (0.110)	−0.303 ** (0.128)
Working place *(Within county = 1, Outside county = 0)*	−0.186 * (0.110)	−0.234 (0.156)
Public servant *(Yes = 1, Others = 0)*	−0.195 (0.126)	−0.438 *** (0.117)
Working in industry or manual labor *(Yes = 1, Others = 0)*	−0.096 (0.227)	−0.238 (0.180)
Self-employed *(Yes = 1, Others = 0)*	−0.113 (0.129)	−0.265 ** (0.120)
Net total income *(1000 RMB/year)*	6.31 × 10^−5^ (6.08 × 10^−5^)	7.70 × 10^−5^ (6.23 × 10^−5^)
**Social and demographic characteristics**		
Ethnic majority (Han) *(Yes = 1, No = 0)*	−0.841 * (0.437)	−1.220 ** (0.477)
Party membership *(Yes = 1, No = 0)*	−0.165 ** (0.077)	−0.169 (0.107)
Hukou (registered permanent residence) *(Resident Non-farmer = 1, Farmer = 0)*	−0.169 ** (0.08)	−0.205 ** (0.100)
**Environmental characteristics**		
Waste classification *(Yes = 1, No = 0)*	−0.079 (0.131)	−0.013 (0.093)
Toilet Revolution Project participation *(Yes = 1, No = 0)*	0.148 (0.160)	0.177 (0.125)
Household toilet sanitation *(1 = least sanitary, … 5 = most sanitary)*	0.260 *** (0.077)	0.167 ** (0.072)
Domestic livestock *(Yes = 1, No = 0)*	−0.056 (0.108)	−0.005 (0.114)
Livestock waste *(emission < 500 g/day)*	0.058 (0.080)	0.050 (0.097)
Livestock waste *(emission = 500–1000 g/day)*	0.162 (0.262)	0.026 (0.194)
Livestock waste *(emission > 1000 g/day)*	−0.163 (0.118)	−0.208 (0.132)
**Village characteristics**		
Number of workers returning to the village from the outside province *(persons)*	0.001 (0.001)	0.001 (0.001)
Number of workers returning to the village from the outside city *(persons)*	−0.007 *** (0.002)	−0.005 *** (0.001)
Number of workers returning to the village from the outside county *(persons)*	0.001 (0.001)	−0.005 (0.007)
Main source of funding for Toilet Revolution *(Government = 1, Self = 0)*	0.170 * (0.094)	0.217 ** (0.103)
Disposal of toilet waste *(Properly treated = 1, Not properly treated = 0)*	0.233 ** (0.109)	0.290 *** (0.104)
Time spent by village leaders on dealing with village affairs *(Hours/Day)*	0.016 *** (0.005)	0.016 *** (0.006)
Maintenance cost of water conservancy infrastructure *(RMB/Average year)*	9.93 × 10^−7^ (2.53 × 10^−6^)	4.87 × 10^−6^ ** (2.27 × 10^−6^)
Wage of male workers *(RMB/day)*	0.002 *** (0.001)	0.002 ** (0.001)
Wage of female worker *(RMB/day)*	−0.002 (0.001)	−0.001 (0.001)
Pseudo-R^2^	0.046	0.063
Observations	1064	1064

Notes: The values outside parenthesis are the regression coefficients, showing the size and direction of association between the dependent and independent variables. The values inside the parenthesis are the corresponding standard errors from the regression results. * Significant at the 10% level, ** significant at 5%, and *** significant at 1%.

## Data Availability

Th data supporting reported results can be obtained from the corresponding author upon request.

## Appendix A

### Appendix A.1. Descriptive Statistics of the Model Variables

Figure A1 reports descriptive statistics for respondents’ satisfaction with waste disposal management services and waste collection and disposal infrastructure. An almost similarly high proportion (~82%) reported that they are ‘satisfied’ with both waste disposal management services (WDMS) and waste collection and disposal infrastructure (WCDI). More than 12% of the respondents were highly satisfied, while only a few (0.09–0.38%) were very dissatisfied with WDMS and WCDI in the study area.

Table A1 shows descriptive statistics for the explanatory variables used in this study. Only around one quarter (27%) of the respondents reported paying some fee for waste disposal services in their village. Regarding waste disposal methods, more than three-quarters of the respondents reported throwing their household solid waste in public waste bins, in public storage buildings, in public pits, with only very few (1.4%) reporting littering the waste, suggesting that the majority of the respondents have high adoption of available waste management services in the area.

For other independent variables, the sample was predominantly male (95.1%). The mean age of the household head (the respondent) was 58.4 years, with a standard deviation of 10.8. Regarding education level, the average years of formal education were 6.5, with a standard deviation of 3.2, indicating that most respondents were educated but with education achievements falling in and around primary school education. In terms of political activism, less than a quarter (18.8%) belonged to the Communist Party of China. The average household’s total net income was 74 thousand RMB, with significant variations among the respondents (high standard deviation). Minorities make up a tiny (0.7%) of the sample. Around three-quarters (72.3%) of respondents reported holding farmer hukou, suggesting that a significant portion was landless rural residents (Resident Non-farmer). The majority (84%) of respondents reported having mobile phone internet, showing the popularity and diffusion of the internet and mobile technology in the area. Most respondents were self-employed (54%) or professionals in public service (31%).

Under the environmental variables, only a small portion of respondents (10.7%) indicated that they were involved in classifying/sorting their household waste. One-tenth of respondents also participated in some skills training programs organized by the government. Regarding household toilet sanitation, 61.2% and 23.3% of sampled people reported their toilets as moderately and more sanitary, respectively. A significant proportion of respondents did not have livestock waste emissions (45.6%) or low emissions (42.3%).

The highest number of workers returning to the village from other places was outside the province (17). Over 81% of respondents reported that government funding was the primary source of the Toilet Revolution Project funds in their village. Similarly, 84% of respondents reported that the village authorities used appropriate methods to dispose of toilet waste in their village. The village leadership cadre was reported to have spent over 11 h daily dealing with village affairs. The average male worker’s wage was 308 RMB, much higher than the female wage of 238 RMB.

**Table A1:** Descriptive analyses.

Variable	Definition	%	Mean	SD
Independent variable				
Waste payment	Yes = 1, No = 0	27.1		
Waste disposal methods				
Method 1	Littering = 1, Others = 0	1.4		
Method 2	Door-to-door collection = 1, Others = 0	4.6		
Method 3	Method 2 + Storing = 1, Others = 0	17.7		
Method 4	Thrown in public waste bins, in public storage buildings, in public pits = 1, Others = 0	76.4		
Gender	Male = 1, Female = 0	95.1		
Age	Number of years		58.4	10.8
Education	Number of years of education		6.5	3.2
Party membership	Yes = 1, No = 0	18.8		
Net total income	1000 RMB/year		74.0	685.6
Ethnic majority (Han)	Han ethnicity = 1, Non-Han ethnicity = 0	99.3		
Hukou (registered permanent residence)	Resident Non-farmer = 1, Farmer = 0	27.7		
Waste classification	Yes = 1, No = 0	10.7		
Skills training	Yes = 1, No = 0	10.6		
Mobile internet	Yes = 1, No = 0	84.2		
Toilet Revolution Project participation	Yes = 1, No = 0	10.5		
Household Toilet Sanitation				
Very unsanitary		4.4		
Unsanitary		11.0		
Moderately sanitary		61.2		
More sanitary		23.3		
Very sanitary				
Domestic livestock	Yes = 1, No = 0	57.6		
Livestock waste emission (g/day)				
Category 1	None = 1, Others = 0	45.6		
Category 2	>0 <500 g = 1, Others = 0	42.3		
Category 3	500–1000 g = 1, Others = 0	4.8		
Category 4	>1000 g = 1, Others = 0	7.3		
Working place	Work within county = 1, Work outside county = 0	82.1		
Profession				
Agriculture	Agriculture = 1, Others = 0	10.0		
Public servant	Public servant = 1, Others = 0	30.9		
Working in industry or manual labor	Working in industry or manual labor = 1, Others = 0	4.2		
Self-employed	Self-employed = 1, Others = 0	54.9		
Number of workers returning to the village from the outside province	Number of persons		17.2	49.2
Number of workers returning to the village from the outside city	Number of persons		3.2	14.1
Number of workers returning to the village from the outside county	Number of persons		2.2	7.0
The main source of funding for the Toilet Revolution Project	Government funding = 1, self-financing = 0	81.4		
Disposal method of waste from household toilet	Properly treated = 1, Not properly treated = 0	84.3		
Time spent by village Party Secretary and Village Director on dealing with village affairs	Hours/Day		11.5	4.9
Maintenance cost of water conservancy infrastructure	RMB/Average year		9524.3	19,155
Wage of male workers	RMB/day		308.0	94.8
Wage of female workers	RMB/day		234.7	72.8

Source: variables are based on our survey data.

### Appendix A.2. Socio-Economic Background of Sampled Villages

The average population in sampled villages was 1966, with a 7729 RMB/year per capita income. Non-agricultural activities accounted for about two-thirds of the total income (66%). The average labor force size (aged 16–65) was 1126 persons per village. For agricultural resources, the average size of the cultivated land was 127.4 ha, where the land can be leased at 4140 RMB/year rent. More than 64 percent of the villages reported having organized at least one skills training program for their residents in the previous year.

Regarding basic infrastructure, around 46% of villages had at least one public toilet. The average distance from the nearest national highway was 2.8 km, with more than 11 public transport shuttles running through the village daily. An average village was 11.8 km away from the village committee seat of the area.

**Table A2:** Socio-economic background of villages in the sample.

	%	Average	SD
Population (people)		1965.5	1228.2
Average yearly income (RMB/capita)		7728.6	4144
Ratio of non-agricultural in total income		66	23.4
Total labor force (16–65 years old)		1126.3	790.2
Cultivated land (ha)		127.4	116.2
Agricultural land rent (RMB/ha/year)		4140.0	3210.0
Organized at least one skills training in the last year	64.5		
Village has at least one public toilet	45.8		
Distance from nearest national highway (km)		2.8	7.9
Number of public bus shuttles/day		11.5	12.5
Distance from Village Committee Seat		17.8	19.8

Source: variables are based on our survey data.
